# Lsm1 Coordinates Mitochondrial Homeostasis, TORC1 Signaling, and Virulence in *Candida albicans*

**DOI:** 10.3390/microorganisms14040771

**Published:** 2026-03-28

**Authors:** Hangqi Zhu, Jianing Wang, Lin Liu, Qilin Yu, Mingchun Li

**Affiliations:** National Key Laboratory of Intelligent Tracking and Forecasting for Infectious Diseases, College of Life Sciences, Nankai University, Tianjin 300071, China; 1120220618@mail.nankai.edu.cn (H.Z.); 2120241655@mail.nankai.edu.cn (J.W.); 2120231466@mail.nankai.edu.cn (L.L.)

**Keywords:** processing body, Lsm1, TORC1, mitochondrion, *Candida albicans*

## Abstract

The fungal pathogen *Candida albicans* coordinated metabolism, organelle homeostasis, and stress responses for adapting to diverse host environments and maintaining virulence. While transcriptional control of these processes has been extensively studied, the contribution of post-transcriptional regulation remains incompletely understood. Here, we identify the P-body component Lsm1 as a critical factor of metabolic adaptation, mitochondrial homeostasis, and pathogenicity in *C. albicans*. Transcriptomic analysis revealed that loss of Lsm1 causes global transcriptional imbalance, leading to dysfunction of amino acid metabolism, mitochondrial function, endocytic trafficking, and autophagy processes. This dysfunction is accompanied by diminished TORC1 activity. Due to the aberrant TORC1 regulation caused by loss of Lsm1, *ATG* mRNA stability and autophagy flux was impaired under nutrient-rich condition and nitrogen starvation condition. In this context, the *lsm1*Δ/Δ cells established an adaptive metabolic and redox state characterized by altered NAD^+^/NADH and NADP^+^/NADPH balance, and enhanced antioxidant capacity. Moreover, the *lsm1*Δ/Δ cells displayed the defects in hyphal development, biofilm formation, and host cell interaction, and exhibited the attenuated virulence in a murine infection model. Together, our findings revealed that Lsm1-mediated post-transcriptional regulation is associated with the maintenance of amino acid metabolism, mitochondrial function, and TORC1 activity to fungal virulence, revealing a potential therapeutic target for *C. albicans* infections.

## 1. Introduction

*Candida albicans* is a major opportunistic fungal pathogen of humans that occupies a wide range of divergent niches within the host, including the skin, oral cavity, gastrointestinal and genitourinary tracts [1,2,3]. Encountering diverse hostile stresses within the host microenvironment, the ability to transition between commensal and pathogenic states relies on flexible coordination between metabolic shift, organelle homeostasis, and stress adaptation [4,5]. This critical evolutionary strategy is associated with its pathogenicity, yet it constitutes a formidable challenge to human health. Moreover, *C. albicans* was classified as a human fungal pathogen of critical priority by the World Health Organization (WHO) [6,7].

In eukaryotes, mitochondria serve not only as bioenergetic centers but also as hubs for redox balance, metabolic regulation and signal transduction [8,9]. The mitochondrial network contains distinct regions that perform specialized functions [10,11]. For instance, the endoplasmic reticulum–mitochondria encounter structure (ERMES) is involved in regulating lipid exchange, calcium signaling, mitochondrial fission, and mitochondrial DNA replication [12,13]. In the recent years, it has been reported that processing bodies (P-bodies; PBs), membrane-less cytoplasmic condensates conserved from yeast to human, are associated with mitochondria [14,15,16]. This organelle participates in modulating mRNA decay, acting as sites of RNA storage during cellular stress, and translation repression [17,18]. Although much is known about the formation of PBs involving liquid–liquid phase separation through multiple protein–protein and protein–RNA interactions [19,20], PBs’ interaction with other cytoplasmic organelles remains unclear. In budding yeast, the Pumilio family member Puf3 has been reported to accumulate in P-bodies and mitochondria-associated foci [21,22]. Puf3 preferentially binds the 3′ UTR of mRNAs of nuclear-encoded mitochondrial proteins, which contributes to their localization at the periphery of the mitochondria and to their deadenylation and degradation [23]. However, P-bodies contain numerous protein components. Puf3 is not a high-concentration core component of PB. Thus, whether the core components of PB directly participate in regulating mitochondrial structure and function still requires further investigation.

The Target of Rapamycin (TOR) signaling pathway, as a central hub, senses intracellular nutrient availability and physical environment, including amino acid, and regulates cellular growth, stress adaptation and responses to host cells [24,25]. Importantly, mitochondrial function and TORC1 activity are highly interdependent: mitochondrial metabolism influences amino acid pools and redox balance, while TORC1 signaling reciprocally regulates mitochondrial biogenesis, respiratory activity, and quality control [26,27]. In addition, autophagy acts downstream of TORC1 to remove damaged organelles and maintain cellular fitness under fluctuating environmental and host-associated stresses [24]. In yeast, studies have shown that the P-body components, including the Pat1-Lsm complex, can post-transcriptionally regulate autophagy-related (*ATG*) genes under nitrogen starvation conditions, and another component Dhh1 plays a role in the effective translation of Atg1 and Atg13 [28,29]. Consequently, disruption of the coordinated interplay between mitochondria, TORC1 signaling, and autophagic quality control is expected to impair cellular adaptation and attenuated virulence in pathogenic fungi. Whether P-body core components impact the virulence and pathogenicity of *Candida albicans* by regulating this network remains to be explored.

Lsm1 is a core constituent of P-bodies and a conserved component of the Lsm1-7 complex, primarily implicated in mRNA decapping and 5′-3′ mRNA decay in *Saccharomyces cerevisiae* [30]; its role in *C. albicans* remains to be identified. Here, we show that a population of PBs are associated with mitochondria in *C. albicans*. Deletion of *LSM1* disrupts amino acid metabolism, induces mitochondrial dysfunction, and reduces TOR activity and uncouples autophagy initiation from degradative flux. Importantly, these defects induce metabolic reprogramming and adaptive redox responses, ultimately leading to profound attenuation of virulence in vitro and in vivo. Our findings establish a critical link between post-transcriptional regulation of metabolic shift, mitochondrial function and virulence in fungal pathogen *C. albicans*, potentially unveiling novel therapeutic avenues and strategies for combating candidiasis.

## 2. Materials and Methods

### 2.1. Strains and Culture Conditions

The strains and plasmids used in this study are listed in Supplementary Table S1. All *Candida albicans* strains were cultured in liquid YPD medium (1% yeast extract, 2% peptone, and 2% glucose) at 30 °C with shaking at 160 rpm to logarithmic phase. Further experimental conditions are specified otherwise. Nitrogen starvation was induced by transferring to SD-N medium (1.04% (*m*/*v*) MgSO_4_·7H_2_O, 3.04% (*m*/*v*) KH_2_PO_4_, 1.04% (*m*/*v*) KCl, 0.1% (*v*/*v*) 1000 × trace element solution, 0.1% (*v*/*v*) 1000 × vitamin solution). To induce mitochondrial oxidative stress, fungal cells in logarithmic phase were treated with rotenone (10 μg/mL, MCE, Monmouth Junction, NJ, USA) for 1 h at 30 °C with shaking at 160 rpm before being harvested for subsequent assays.

Strain manipulations were performed using standard methods and culture conditions. Gene deletion and tagging were performed by homologous recombination and confirmed by PCR on genomic DNA. To select *C. albicans* transformants, cells were plated in SD selective medium (glucose 2%, yeast nitrogen base 0.67%, amino acid drop-out mixture 0.2%, and agar 2%) lacking uracil or histidine, and colonies were checked by PCR and sequencing. The primers used for the construction of the mutants are listed in Supplementary Table S2.

### 2.2. Transcription Profiling Analysis

To investigate the effect of *LSM1* deletion on transcription profiling, the strains WT and *lsm1*Δ/Δ were cultured in liquid YPD medium at 30 °C for 4 h, and then the cells were harvested for RNA extraction. Three independent biological replicates were prepared for each strain. RNA concentration and purity was analyzed using NanoDrop 2000 spectrophotometer (Thermo Fisher Scientific, Waltham, MA, USA). RNA integrity was assessed using the RNA Nano 6000 Assay Kit of the Agilent Bioanalyzer 2100 system (Agilent Technologies, Santa Clara, CA, USA). Sequencing libraries were generated using NEBNext UltraTM RNA Library Prep Kit for Illumina (NEB, Ipswich, MA, USA) following manufacturer’s recommendations. Raw sequencing reads were subjected to quality control using FastQC, and low-quality reads and adaptor sequences were removed before downstream analysis. Gene annotations were retrieved from *C. albicans* genome browser (www.candidagenome.org). Differential expression analysis was conducted using the DESeq2, and statistical significance was evaluated using adjusted *p*-values calculated by the Benjamini–Hochberg false discovery rate (FDR) correction.

### 2.3. Spot Assays

To test the growth of *C. albicans* cells in the medium (containing a respiratory carbon source), the overnight cultured strains were diluted to OD_600_ = 0.1, grown to exponential phase. The strains were harvested and diluted to OD_600_ = 0.2. Then the 10-fold serial dilutions were spotted on solid SC medium containing 2% glucose, 3% glycerol or 2% ethanol. For the assay of oxidative stress, the 10-fold serial dilutions were spotted on solid YPD medium with or without 5 mM H_2_O_2_. The plates were incubated at 30 °C for 2–3 days and photographed.

### 2.4. Western Blotting

The samples were prepared in lysis buffer (50 mM Tris-HCl pH 7.4, 150 mM NaCl,1% NP-40, 1% sodium deoxycholate, 0.1% SDS, 1 mM EDTA, 1 mM PMSF, 1:100 Protease Inhibitor Cocktail). Samples were loaded on SDS-PAGE or Phos-tag SDS-PAGE and analyzed by Western blotting. GFP-Atg8 fusion proteins were detected with monoclonal anti-GFP antibody (MBL, Tokyo, Japan, 1:3000 dilution), Sch9-HA fusion proteins were detected with monoclonal anti-HA antibody (Sigma, St. Louis, MO, USA, 1:3000 dilution), and GAPDH was detected using anti-GAPDH antibody (ZENBIO, Chengdu, China, 1:5000 dilution). HRP-conjugated goat anti-mouse IgG (BioRad, Hercules, CA, USA, 1:5000 dilution) was used as the secondary antibody. Phos-tag SDS-PAGE was performed using a gel prepared with 8% (*w*/*v*) polyacrylamide, Phosbind acrylamide (20 μM), 2 equivalents of MnCl_2_, 375 mM Tris-HCl (pH 8.8) and 0.1% SDS at 30 mA/gel for 60 min at room temperature. The electrophoresis running buffer (pH 8.4) was 25 mM Tris, 192 mM glycine and 0.1% SDS.

### 2.5. Real-Time qPCR (RT-qPCR)

Strains cultured to logarithmic phase in liquid YPD (30 °C, 160 rpm) were used as the nutrient-rich control. For the nitrogen starvation group, cells were harvested and resuspended in SD-N medium for 1 h. Total RNA was extracted using RNA extraction kits (Promega, Madison, WI, USA), and the cDNAs were prepared from the extracted RNAs using TransScript Uni All-in-One First-Strand cDNA Synthesis Kits (TransGen, Beijing, China). The expression of interested genes was detected by using the RealMaster Mix (SYBR Green) Kit (TransGen, Beijing, China). RT-qPCR analysis was performed by using the IQTM5 Multicolor Real-Time PCR Detection System (BIO-RAD, Hercules, CA, USA). Transcription levels of these genes were normalized against the levels of *ACT1*. Relative expression levels were calculated using the 2^−ΔΔ*Ct*^ method from three independent biological replicates, each performed in technical triplicate.

### 2.6. Confocal Microscopy

For Lsm1 localization analysis, the strain WT-GFP-Lsm1 stained with Hoechst 33342 (5 μg/mL) and MitoTracker Deep Red FM (0.1 mmol/L, prepared in DMSO, Invitrogen, Carlsbad, CA, USA) for 30 min was observed by confocal microscopy with a 63× oil-immersion objective. (Zeiss LSM710, Oberkochen, Germany).

### 2.7. Assay of Mitochondrial Membrane Potential

To test the mitochondrial membrane potential, cells were cultured overnight in YPD medium at 30 °C with shaking, adjusted to an OD_600_ of 0.1 in YPD and cultured for 4 h. Cells were collected and stained by JC-1 (5 μM, prepared in DMSO, Sigma, USA) for 30 min at 30 °C. After washed twice with PBS, the cells were assessed by flow cytometry (FACS Calibar flow cytometer, BD, Sparks, MD, USA) to evaluate the mitochondrial membrane potential. Depolarized mitochondria were detected as JC-1 monomers (green fluorescence, Ex 488 nm/Em 530 nm), whereas healthy, hyperpolarized mitochondria were characterized by JC-1 J-aggregates (red fluorescence, Ex 488 nm/Em 590 nm). A minimum of 10,000 events were acquired per sample.

### 2.8. Measurement of Intracellular ROS and Calcium Levels

Intracellular ROS was detected using the oxidant-sensitive agent DCFH-DA (10 μM, Sigma, USA). The cells were stained for 30 min at 30 °C and washed twice with PBS. Then, the fluorescence density (FLU) was detected by a fluorescence microplate reader (Agilent, USA). Cells were also counted using a spectrophotometer with OD_600_. The relative fluorescence density of each sample was calculated as FLU divided by OD_600_ to evaluate intracellular ROS levels. Intracellular calcium levels were determined following the same procedure, using the calcium-sensitive dye Fluo-4 AM (2 μM, MCE, USA) for cytosolic Ca^2+^ and the mitochondria-targeted indicator Rhod-2 AM (10 μM, MCE, USA) for mitochondrial Ca^2+^ levels.

### 2.9. Assay of NAD^+^/NADH and NADP^+^/NADPH

The intracellular levels of NAD^+^/NADH were quantified using commercial assay kits according to the manufacturer’s instructions with minor modifications (CheKine^TM^ Micro Coenzyme I NAD(H) Assay Kit, Abbkine, Wuhan, China). Cells were grown in YPD medium to logarithmic phase, harvested by centrifugation at 4000× *g* for 5 min at 4 °C, and washed twice with ice-cold PBS. Cell pellets (≈2 × 10^8^ cells) were resuspended in 300 μL NAD^+^/NADH extraction buffer provided in the kit (modified from the manufacturer’s protocol in terms of starting cell number and extraction volume). The amount of starting material and extraction volume were optimized in preliminary experiments to ensure that the measured absorbance values fell within the linear range of the standard curve. Cells were disrupted by vortexing with acid-washed glass beads (30 s vortexing followed by cooling on ice, repeated 8–10 times) (modification: bead-beating was used instead of the ultrasonic disruption recommended in the kit protocol). The lysates were boiled for 5 min to inactivate enzymes and subsequently neutralized by adding 20 μL Assay Buffer and 100 μL of the complementary extraction buffer (NADH extraction buffer for NAD^+^ determination or NAD extraction buffer for NADH determination). Samples were centrifuged at 13,000× *g* for 10 min at 4 °C, and the supernatants were collected for analysis. An amount of 40 μL of sample extract was added to a 96-well microplate and mixed with 80 μL of freshly prepared reaction working solution containing Assay Buffer, NAD cycling enzyme mix, WST-8, enhancer, and ethanol solution as specified by the kit instructions. After incubation at room temperature for 30 min, absorbance at 450 nm was measured using a microplate reader.

The intracellular levels of NADP^+^/NADPH were quantified using commercial assay kits according to the manufacturer’s instructions with minor modifications (NADP^+^/NADPH Assay Kit with WST-8, Beyotime, Shanghai, China). Cells were harvested and lysed in NADP^+^/NADPH extraction buffer provided in the kit. After vigorous vortexing with glass beads, the lysates were centrifuged at 13,000× *g* for 10 min at 4 °C, and the supernatants were collected as sample extracts. To distinguish oxidized and reduced forms, each sample was split into two aliquots: one was used directly to determine total NADP(H), while the other was incubated at 60 °C for 30 min to selectively decompose NADP^+^ for measurement of NADPH. After cooling and centrifugation to remove precipitates, the supernatants were used for subsequent assays. For the colorimetric reaction, 20 μL of sample extract was added to a 96-well plate followed by 90 μL alcohol dehydrogenase working solution. After incubation at 37 °C for 10 min, 10 μL WST-8 chromogenic solution was added and the reaction was further incubated at 37 °C for 20 min in the dark. Absorbance at 450 nm was measured using a microplate reader. NADP^+^ levels were calculated by subtracting NADPH from total NADP(H), and the NADP^+^/NADPH ratio was determined accordingly. The results were normalized to total protein concentration measured by the BCA assay.

### 2.10. Assay of Superoxide Dismutase (SOD) Activity

SOD activity was determined using a commercial Superoxide Dismutase Assay Kit with WST-8 (Beyotime) according to the manufacturer’s instructions. Briefly, 20 μL of sample lysate was mixed with 200 μL of WST-8/enzyme working solution in a 96-well plate, incubated at 37 °C for 20 min, and the absorbance at 450 nm was recorded using a microplate reader. One unit of SOD activity was defined as the amount of enzyme needed to exhibit 50% inhibition of the WST-8 reaction under assay conditions. Enzyme activities were normalized to total protein content measured in parallel by the BCA assay.

### 2.11. Assay of GSH/GSSG

The well-cultured fungal cells were harvested by centrifugation and washed twice with PBS. The weight of the cell pellet was determined by weighing the centrifuge tube before and after cell collection, and the difference was calculated. Three volumes of extraction solution from a commercial GSH and GSSG Assay Kit (Beyotime Biotechnology, Shanghai, China) relative to the cell pellet were added to the samples. Cells were disrupted using 6–8 rapid freeze–thaw cycles by alternating between liquid nitrogen and a 37 °C water bath. The lysates were then incubated on ice for 5 min and centrifuged at 10,000× *g* for 10 min at 4 °C. The supernatants were collected for determination of total glutathione. For GSSG measurement, an aliquot of the prepared sample was treated with GSH-depleting auxiliary solution (20 µL per 100 µL sample) and the GSH-depleting working solution (4 µL per 100 µL sample). The mixture was vortexed immediately and incubated at 25 °C for 60 min to remove GSH. The resulting samples were subsequently used for GSSG determination according to the manufacturer’s instructions.

### 2.12. Assay of Hyphal Growth and Biofilm Formation

RPMI-1640 and Spider solid media were used to induce hyphal growth. The overnight cultured strains were diluted in sterile water to an OD_600_ of 0.5, dotted on the solid plates. The plates were incubated at 37 °C for 3–5 days and photographed. To induce embedded growth, cells were cultured to the exponential phase, washed, mixed in molten YPD semi-solid medium (1% agar) and incubated at 25 C for 3–5 days. RPMI-1640 was used for liquid induction of morphogenetic switching. The strains were diluted in RPMI-1640 to an OD_600_ of 0.05, shaken at 37 °C for 3 h or 6 h and collected for microscopic observation. For the biofilm formation assay, cells were incubated on polystyrene plates at 37 °C for 12 h, followed by fixation with 2.5% (*v*/*v*) glutaraldehyde at 4 °C for 4 h. After washing with PBS, the samples were dehydrated through a graded series of ethanol (30%, 50%, 70%, 80%, 90%, and 100% for 20 min each). The samples were then lyophilized in vacuum desiccators. After being sputter-coated with gold, the samples were observed by scanning electron microscopy (FEI QUANTA 200, Thermo Fisher Scientific, Waltham, MA, USA).

### 2.13. In Vitro Fungus-293T Cell Interaction Model

The human embryonic kidney cell line 293T was procured from the Cell Resource Center (China Academy of Medical Science, Beijing, China). The 293T cells were cultured in DMEM medium supplemented with 10% FBS in 24-well polystyrene plates in a humidified incubator at 37 °C with 5% CO_2_ for 2 days. Cell monolayers in each well were washed twice with PBS to remove any residual media. The cells were then incubated with 5 × 10^5^ log-phase fungal cells in 500 μL of the 293T culture medium and incubated at 37 °C for 2 h. After incubation, the samples underwent the same fixation, dehydration and SEM preparation steps as the biofilm assay. The percentages of adhesion, invasion into 293T cells, and hyphal reorientation in the strains were determined as previously described [31].

### 2.14. In Vivo Fungal Infection Assays

For virulence assay, overnight cultures of the corresponding strains were harvested and re-suspended in a 0.9% NaCl solution to create a standardized inoculum. Female ICR mice, aged 5–6 weeks, were used for the assay. Each mouse was inoculated with 5 × 10^6^ *C. albicans* cells via the tail vein to simulate systemic infection. The survival rate of the mice was monitored over a 20-day period to assess the virulence of the fungal strains and the progression of the infection. After 5 days post-inoculation, three mice from each group were sacrificed to determine the fungal burden in the kidneys, a common target organ for *Candida* species. The kidneys were removed, homogenized to disrupt tissue and release fungal cells, and then serial dilutions were prepared. The diluted homogenates were plated on YPD agar plates to culture and enumerate the viable fungal cells. For histopathological analysis, the kidneys from the sacrificed mice were fixed, processed, and embedded in paraffin to create tissue sections. The sections were stained with hematoxylin and eosin (H&E); then, the stained sections were observed under a light microscope (Leica DM3000, Wetzlar, Germany) to assess tissue damage, inflammation, and fungal invasion.

### 2.15. Statistical Analysis

Differences between two groups were analyzed using Student’s *t*-test, and comparions among multiple groups were performed using one-way ANOVA followed by Tukey’s multiple comparison test. For all analyses, a *p*-value of less than 0.05 (*p <* 0.05) is considered statistically significant. The data are presented as means ± standard deviations (s.d.) based on three or more independent experiments.

## 3. Results

### 3.1. RNA Sequencing of lsm1Δ/Δ Revealed Strong Changes in Mitochondrial Function and Energy Metabolism

**Figure 1:**
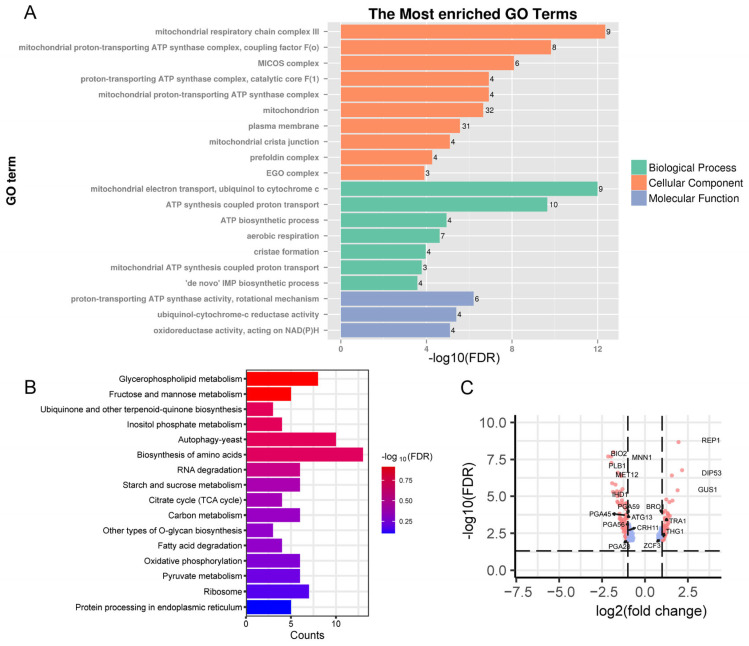
Loss of Lsm1 induces global transcriptional imbalance in *C. albicans*. (**A**) GO enrichment analysis showing significant enrichment of mitochondrion-associated pathways. (**B**) KEGG pathway analysis of differentially expressed genes in *lsm1*Δ/Δ relative to wild type. (**C**) Volcano map of differentially expressed genes. Statistical significance was evaluated using adjusted *p*-values calculated by the Benjamini–Hochberg false discovery rate (FDR) correction. The x-axis represents log_2_ (fold change) in gene expression between the two groups, and the y-axis represents −log_10_ (FDR). Red dots indicate significantly upregulated genes, and blue dots indicate significantly downregulated genes. The horizontal dashed line indicates the significance threshold (FDR < 0.05), and the two vertical dashed lines represent log_2_(fold change) = 1 and −1.

To determine the global impact of *LSM1* deletion on cellular homeostasis, we compared transcriptomes from WT and *lsm1*Δ/Δ. Prior to differential expression analysis, quality control assessments were conducted to evaluate the consistency and reliability of the sequencing data. Principal component analysis (PCA) based on normalized gene expression profiles showed that biological replicates clustered closely together, while clear separation was observed between the WT and *lsm1*Δ/Δ samples (Figure S1), indicating good reproducibility and minimal batch effects. Differential expression analysis identified 280 differentially expressed genes (105 upregulated genes and 175 downregulated genes). Gene ontology (GO) analysis revealed widespread transcriptional changes in mitochondrial-associated pathways, including mitochondrial respiratory chain complexes, the ATP synthase complex, MICOS complex, and genes involved in mitochondrial crista junction and formation (Figure 1A). KEGG pathway analysis further showed significant enrichment in processes related to amino acid metabolism, fatty acid metabolism, autophagy, RNA degradation, glycerophospholipid metabolism and inositol phosphate metabolism (Figure 1B). RNA sequencing data were visualized as a volcano plot, highlighting that genes with the highest q-values and fold-change values were related to amino acid metabolism (i.e., *MET12*, *DIP53*), biotin synthesis (i.e., *BIO2*), tRNA processing (i.e., *GUS1*, *THG1*), cell wall integrity (i.e., *CRH11*, *IHD1*, *PGA28*, *PGA45*, *PGA56*, *PGA59*) and interspecies interaction (i.e., *MNN1*, *PLB1*), response to nutritional signals or biotic stimulus (i.e., *REP1*, *ZCF3*) and autophagy (i.e., *ATG13*) (Figure 1C). Taken together, the transcription profiling suggests that Lsm1 plays an important role in maintaining transcriptional programs required for mitochondrial integrity and metabolic balance.

### 3.2. Dysregulation of Amino Acid Biosynthesis and Transport Genes in lsm1Δ/Δ

**Figure 2:**
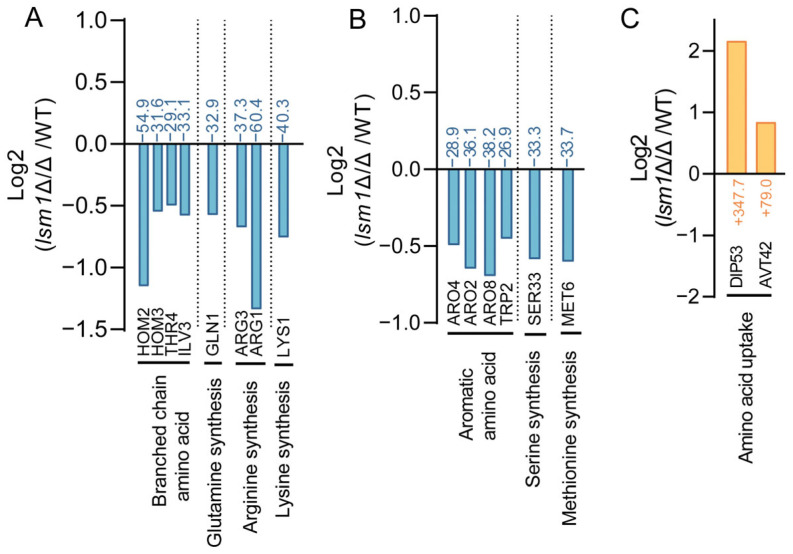
Transcriptional changes in amino acid metabolism upon *LSM1* Deletion. (**A**,**B**) Relative expression levels of genes involved in amino acid synthesis between WT and *lsm1*Δ/Δ, presented as Log_2_ fold change and the percentage of differentially expressed genes. Amino acid synthesis genes are grouped according to different kinds of amino acid metabolism. (**C**) Relative expression levels of genes involved in amino acid transport between WT and *lsm1*Δ/Δ, shown as Log_2_ fold change and the percentage of differentially expressed genes. Color of genes indicate the direction and statistical significance of expression changes: significant downregulation (*p <* 0.05, blue), significant upregulation (*p <* 0.05, orange). Genes involved in amino acid synthesis and uptake with significant transcriptional changes are shown. The dashed lines in the figure serve as visual separators for different functional group of genes.

Multiple genes involved in amino acid biosynthesis and metabolism were transcriptionally downregulated in *lsm1*Δ/Δ (Figure 2, Table S3), suggesting impaired endogenous amino acid production. The most significant decreases were observed in the synthesis of branched chain amino acid, glutamine, arginine, lysine, aromatic amino acid, serine, and methionine. Meanwhile, genes responsible for amino acid uptake, such as *DIP53* and *AVT42*, were significantly upregulated (Figure 2A–C). These findings suggest that *lsm1*Δ/Δ cells experience a state of amino acid imbalance characterized by increased nutrient uptake but inefficient intracellular utilization or retention.

### 3.3. Deletion of LSM1 Causes Mitochondrial Dysfunction

**Figure 3:**
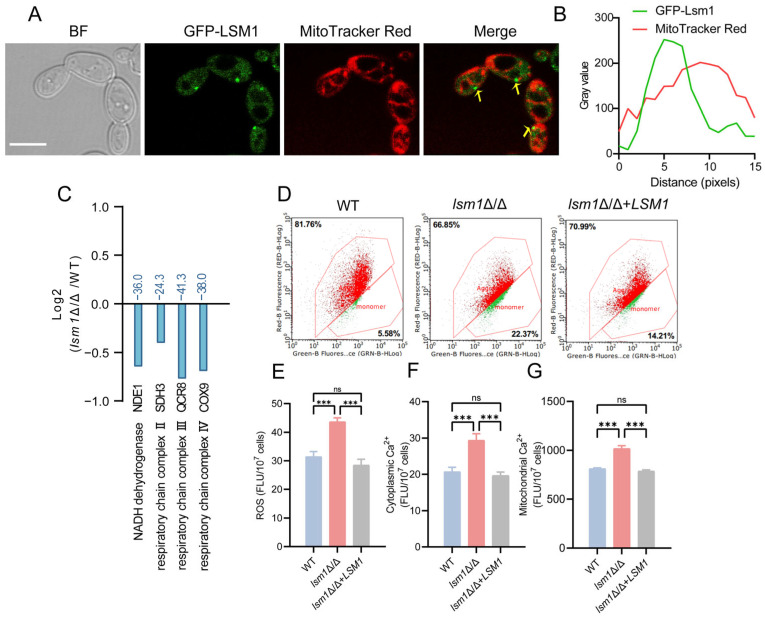
Loss of Lsm1 induces mitochondrial dysfunction. (**A**) Localization of the PB marker GFP-Lsm1 with MitoTracker Red. BF, bright field; GFP, fluorescence showing the localization of GFP-Lsm1; MitoTracker Red, mitochondria staining; Merge, overlay of GFP-Lsm1 and MitoTracker Red signals. Arrows highlight GFP-Lsm1 puncta near mitochondria. Scale bar, 5 μm. (**B**) Fluorescence intensity plot profile analysis (line scan) corresponding to the dashed line in (**A**) using the Image J software (version 1.54f). The spatial overlap of green (GFP-Lsm1) and red (MitoTracker) peaks demonstrates co-localization of P-bodies on the mitochondrial surface. (**C**) Relative expression levels of genes involved in mitochondrial respiratory chain complexes in WT and *lsm1*Δ/Δ cells, shown as Log_2_ fold change and the percentage of differentially expressed genes. Significant downregulation (*p <* 0.05, blue). Genes involved in the mitochondrial respiratory chain with significant transcriptional changes are shown. (**D**) Mitochondrial membrane potential (ΔΨm) was quantified by flow cytometric analysis using the JC-1 in WT, *lsm1*Δ/Δ and complemented (*lsm1*Δ/Δ+*LSM1*) strains. Depolarized mitochondria were detected as JC-1 monomers (green fluorescence, Ex 488 nm/Em 530 nm), whereas healthy, hyperpolarized mitochondria were characterized by JC-1 J-aggregates (red fluorescence, Ex 488 nm/Em 590 nm). The red lines indicate the flow cytometry gates used to distinguish the populations of JC-1 aggregates (upper region) and JC-1 monomers (lower region). (**E**) Intracellular reactive oxygen species (ROS) levels were measured using the fluorescent probe DCFH-DA in WT, *lsm1*Δ/Δ and *lsm1*Δ/Δ+*LSM1* strains. (**F**) Cytosolic Ca^2+^ levels were determined using the calcium-sensitive fluorescent dye Fluo-4 AM. (**G**) Mitochondrial Ca^2+^ levels were assessed using the mitochondria-targeted Ca^2+^ indicator Rhod-2 AM. Statistical significance is indicated as follows: ***, *p <* 0.001; ns, not significant.

In light of previous findings indicating an association between PBs and mitochondria, we sought to determine whether PBs exhibit co-localization with mitochondria in *C. albicans*. Confocal microscopy revealed a spatial overlap of the GFP-Lsm1 with MitoTracker Red, suggesting a potential association between PBs and mitochondrial structures (Figure 3A). To quantitatively evaluate the spatial relationship between PBs and mitochondria, we performed line-scan analysis of the fluorescence signals. The fluorescence intensity plot profiles revealed that the spatial overlap of GFP-Lsm1 and MitoTracker Red peaks demonstrates a high degree of spatial correlation (Figure 3B). These data suggest that Lsm1-positive puncta are not randomly distributed but are specifically docked on the mitochondrial membrane. Transcriptomic alterations in mitochondrial-associated pathways prompted us to assess mitochondrial function in *lsm1*Δ/Δ cells. We found that the expression of genes encoding components of the respiratory chain complex downregulated significantly (i.e., *NDE1*, *SDH3*, *QCR8*, *COX9*), suggesting that *LSM1* deletion caused transcriptional suppression of the mitochondrial respiratory chain (Figure 3C, Table S3). Consistent with these changes, *lsm1*Δ/Δ cells displayed a significant reduction in mitochondrial membrane potential (Figure 3D). Mitochondrial dysfunction was further evidenced by elevated reactive oxygen species (ROS) levels (Figure 3E), and we used Fluo-4 AM to check cytosolic Ca^2+^ levels and measured mitochondrial Ca^2+^ concentration by Rhod-2 AM. Both cytosolic and mitochondrial Ca^2+^ levels were significantly increased in *lsm1*Δ/Δ cells (Figure 3F,G), suggesting calcium overload and disturbed mitochondrial redox homeostasis. These data identify mitochondrial stress and calcium dysregulation as central consequences of *LSM1* deletion.

### 3.4. Altered TORC1 Activity and Impaired Autophagic Flux in lsm1Δ/Δ Under Nutrient-Rich and Nitrogen Starvation Conditions

**Figure 4:**
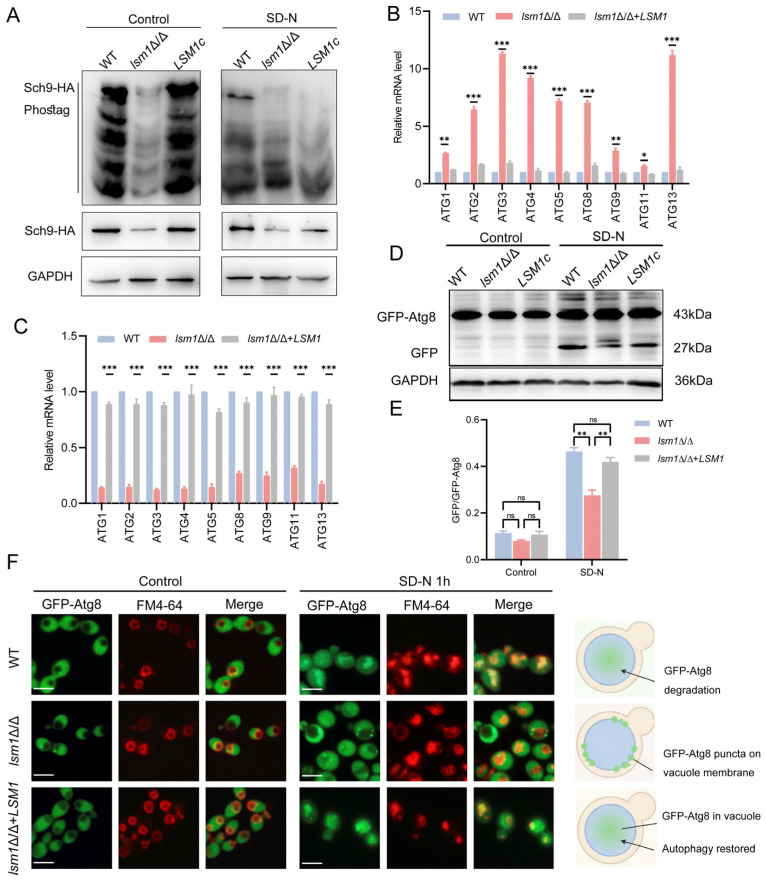
Aberrant TORC1 signaling and impaired autophagy flux in *lsm1*Δ/Δ cells. (**A**) TORC1 activity was assessed by Phos-tag gel electrophoresis of Sch9 phosphorylation in wild-type (WT), *lsm1*Δ/Δ, and complemented (*lsm1*Δ/Δ+*LSM1*) strains under nutrient-rich and nitrogen starvation (SD-N) conditions. (**B**) Relative expression of *ATG* mRNA determined by RT-qPCR under nutrient-rich conditions. (**C**) Relative expression of *ATG* mRNA determined by RT-qPCR under nitrogen starvation (SD-N) conditions. (**D**) Western blot analysis of GFP-Atg8 degradation under nutrient-rich and SD-N conditions. (**E**) Quantification of free GFP levels relative to total GFP-Atg8 by Image J software, indicating autophagic flux efficiency. (**F**) Fluorescence microscopy of GFP-Atg8 localization under nutrient-rich and SD-N conditions. Vacuoles were stained with FM4-64. Scale bar, 5 μm. Data are presented as mean ± SD from at least three independent experiments. Statistical significance is indicated as follows: *, *p <* 0.05; **, *p <* 0.01; ***, *p <* 0.001; ns, not significant.

Since amino acid deficiency and mitochondrial dysfunction are known regulators of TORC1 signaling [32], we next examined whether TORC1 activity was altered under different nutrient conditions. TORC1 activity was assessed by Phos-tag SDS-PAGE to measure the phosphorylated Sch9 protein, a direct TORC1 substrate [32,33]. Under nutrient-rich conditions, Sch9 phosphorylation was markedly reduced in *lsm1*Δ/Δ cells compared to WT and complemented strains, indicating decreased TORC1 activity under basal growth condition (Figure 4A). To further evaluate TORC1 responsiveness to nutrient availability, Sch9 phosphorylation was also examined under nitrogen starvation (SD-N) conditions. As expected, nitrogen starvation led to a pronounced reduction in Sch9 phosphorylation in WT and complemented strains. In contrast, *lsm1*Δ/Δ cells maintained a relatively higher level of Sch9 phosphorylation compared to WT and complemented strains under nitrogen starvation conditions (Figure 4A), suggesting aberrant TORC1 regulation in the absence of Lsm1. The roles of TORC1 signaling in negatively regulating autophagy have been extensively studied. We next examined autophagy-related gene expression and autophagic activity under both nutrient-rich and nitrogen starvation conditions. Notably, previous work has demonstrated that the Pat1–Lsm1-7 complex promotes starvation-induced autophagy by stabilizing ATG mRNAs and protecting them from exosome-mediated degradation during nitrogen starvation in yeast [28]. RT-qPCR analysis revealed that the mRNA levels of multiple ATG genes were significantly elevated in *lsm1*Δ/Δ cells under nutrient-rich conditions (Figure 4B), consistent with reduced TORC1-mediated repression. However, upon nitrogen starvation, ATG mRNA levels in *lsm1*Δ/Δ cells were significantly lower than those in WT and complemented strains (Figure 4C), indicating defective post-transcriptional regulation of ATG transcripts in the absence of Lsm1 in *C. albicans*. To assess autophagic flux, GFP-Atg8 processing was analyzed by Western blot. Under nutrient-rich conditions, GFP-Atg8 degradation was almost undetectable, with no significant differences among strains (Figure 4D,E). In contrast, nitrogen starvation robustly induced GFP-Atg8 cleavage in WT and complemented strains, whereas *lsm1*Δ/Δ cells exhibited a marked reduction in free GFP accumulation, indicating impaired autophagic flux (Figure 4D,E). Consistent with this result, fluorescence microscopy revealed distinct localization patterns of GFP-Atg8. Under nutrient-rich conditions, GFP-Atg8 was diffusely distributed in the cytoplasm in all strains (Figure 4F). Upon nitrogen starvation, GFP-Atg8 localized to FM4-64-stained vacuoles and underwent degradation in WT and complemented strains. In contrast, GFP-Atg8 in *lsm1*Δ/Δ cells failed to enter the vacuolar lumen and instead accumulated as punctate structures along the vacuolar membrane. Together, these results demonstrate that loss of Lsm1 disrupts TORC1 signaling and impairs autophagic flux.

### 3.5. Metabolic Reprogramming and Enhanced Antioxidant Capacity Establish an Adaptive State in lsm1Δ/Δ

**Figure 5:**
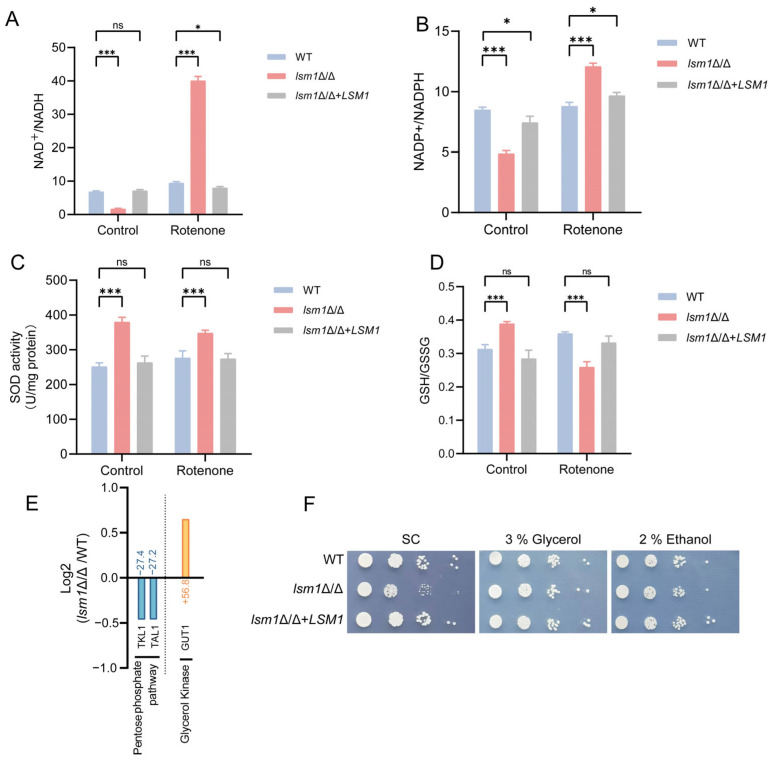
Loss of Lsm1 promotes metabolic reprogramming and antioxidant responses. (**A**) The NAD^+^/NADH ratio of WT, *lsm1*Δ/Δ, and *lsm1*Δ/Δ+*LSM1* strains, with or without rotenone treatment (10 μg/mL). (**B**) The NADP^+^/NADPH ratio of WT, *lsm1*Δ/Δ, and *lsm1*Δ/Δ+*LSM1* strains, with or without rotenone treatment (10 μg/mL). (**C**) Superoxide dismutase (SOD) activity of WT, *lsm1*Δ/Δ, and *lsm1*Δ/Δ+*LSM1* strains, with or without rotenone treatment (10 μg/mL). (**D**) The GSH/GSSG ratio in WT, *lsm1*Δ/Δ, and *lsm1*Δ/Δ+*LSM1* strains, with or without rotenone treatment (10 μg/mL). (**E**) Relative expression levels of genes involved in the pentose phosphate pathway and gene encoding glycerol kinase between WT and *lsm1*Δ/Δ strains, depicted as Log2 fold change and percentages. Color of genes indicate the direction and statistical significance of expression changes: significant downregulation (*p <* 0.05, blue), significant upregulation (*p <* 0.05, orange). Genes involved in other energy metabolism pathways with significant expression changes are shown. (**F**) Growth of the WT, *lsm1*Δ/Δ, and *lsm1*Δ/Δ+*LSM1* strains on media containing 2% glucose, 3% glycerol or 2% ethanol as the carbon source. Statistical significance is indicated as follows: *, *p <* 0.05; ***, *p <* 0.001; ns, not significant.

To characterize the cellular energetic state, we measured the NAD^+^/NADH ratio. Given that NADH serves as a primary electron donor to the mitochondrial respiratory chain, NAD^+^ level was reduced while NADH accumulated in *lsm1*Δ/Δ cells, resulting in a decreased NAD^+^/NADH ratio (Figure 5A), suggesting attenuated electron transport chain activity. In addition, we assessed the NADP^+^/NADPH ratio to evaluate the cellular redox status. Deletion of *LSM1* caused a decrease in the NADP^+^/NADPH ratio (Figure 5B), indicating that the *lsm1*Δ/Δ strain is actively mobilizing its antioxidant defense systems to counteract oxidative stress. The increased activity of superoxide dismutase (SOD) and the elevated GSH/GSSG ratio under basal conditions further suggested a programmed enhancement of antioxidant defenses (Figure 5C,D). Collectively, these findings demonstrate that the cells are experiencing redox imbalance and have mounted an antioxidant defense system to ensure survival.

To test the robustness of this redox state, we challenged the cells with rotenone, a mitochondrial complex I inhibitor [34]. Rotenone treatment rapidly increased the NAD^+^/NADH and NADP^+^/NADPH ratios, reflecting acute consumption of reduced cofactors and suppression of NADH and NADPH generation in *lsm1*Δ/Δ cells (Figure 5A,B). In addition, rotenone treatment significantly decreased SOD activity and lowered the GSH/GSSG ratio in *lsm1*Δ/Δ cells, reflecting the acute consumption of antioxidant reserves and a shift toward an oxidized state (Figure 5C,D). Notably, despite this decline, the SOD activity in rotenone-treated *lsm1*Δ/Δ cells remained higher than that of both WT and complemented strains (Figure 5C). This suggests that while the *lsm1*Δ/Δ mutant’s redox buffer is susceptible to acute stress, its baseline antioxidant capacity is reprogrammed to a higher threshold to compensate for chronic mitochondrial defects.

Additionally, we analyzed the differential gene expression of metabolic pathways in the transcriptome data. The significant downregulation of *TKL1* and *TAL1*, key components of the pentose phosphate pathway, indicates a reduction in carbon flux toward amino acid biosynthetic precursors (Figure 5E). In contrast, the significant upregulation of glycerol kinase *GUT1* suggests that *lsm1*Δ/Δ cells prioritize glycerol utilization as an alternative carbon source to compensate for the energy shortage caused by mitochondrial dysfunction (Figure 5E, Table S3). We next examined whether the increase in glycerol kinase gene expression is reflected in cell growth in media containing respiratory carbon sources. The result showed that the growth situation of *lsm1*Δ/Δ is solved in a medium with 3% glycerol (Figure 5F). The improved growth of the *lsm1*Δ/Δ mutant on ethanol, another non-fermentable carbon source, further demonstrates its slightly enhanced mitochondrial respiratory capacity compared to the WT (Figure 5F). Consequently, the *lsm1*Δ/Δ cells may be switching from a rapid growth state dependent on fermentation and amino acid synthesis to an energy-conserving and respiratory state driven by carbon source utilization. To investigate whether the altered redox balance confers protection, we performed a spot assay under H_2_O_2_ stress. Although the *lsm1*Δ/Δ mutant displayed a modest growth defect on a standard YPD medium, it exhibited enhanced resilience to oxidative stress (Figure S3). These findings indicate that loss of Lsm1 induces metabolic reprogramming and enhanced antioxidant capacity to establish an adaptive redox balance.

### 3.6. Deletion of LSM1 Resulted in Defective Hyphal Growth and Biofilm Formation

**Figure 6:**
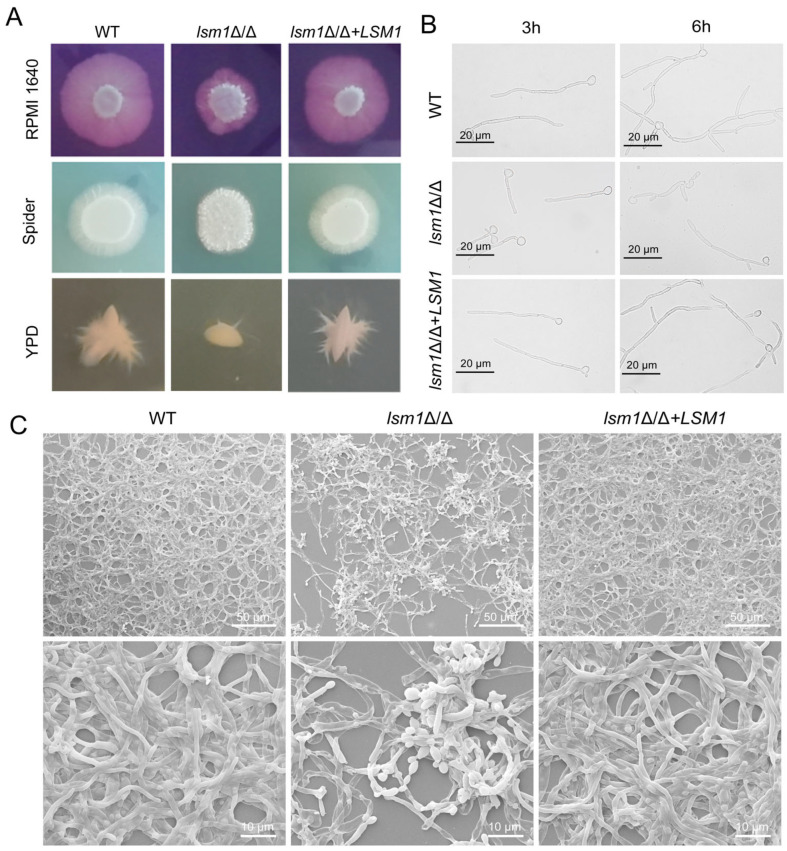
Lsm1 is critical for hyphal growth and biofilm formation in *C. albicans*. (**A**) Hyphal induction in RPMI-1640, Spider solid media and YPD semi-solid medium (1% agar). (**B**) Hyphal induction in liquid RPMI-1640 medium, incubated for 3 h or 6 h. Scale bar, 20 μm. (**C**) Biofilm formation of the WT, *lsm1*Δ/Δ, and *lsm1*Δ/Δ+*LSM1*. Cells were incubated on polystyrene plates at 37 °C for 12 h, lyophilized in vacuum desiccators, and subsequently observed by scanning electron microscopy. The scale bar in the top row of images is 50 μm; the bottom row of images shows an enlarged version of the top row. Scale bar, 10 μm.

Given the extensive metabolic and cellular defects observed in *lsm1*Δ/Δ cells, we examined the impact of Lsm1 loss on pathogenic traits. We found that the *lsm1*Δ/Δ mutant exhibited attenuated filamentation on hypha-inducing plates, including a solid RPMI-1640 medium and Spider medium (Figure 6A). Consistently, the ability of *lsm1*Δ/Δ mutant to grow in an embedded condition was severely impaired (Figure 6A). In a liquid RPMI-1640 medium, the WT and complemented strain *lsm1*Δ/Δ+*LSM1* formed well-developed hyphae after 3 h of incubation, and maintained hyphal growth after 6 h. In contrast, the *lsm1*Δ/Δ mutant displayed a significant reduction in hyphal length compared with the WT and complemented strain *lsm1*Δ/Δ+*LSM1* (Figure 6B, Figure S2). We next explored the effect of Lsm1 on the biofilm formation of *C. albicans* by SEM. The results showed that the biofilm formation of the *lsm1*Δ/Δ mutant was significantly reduced compared with the WT and *lsm1*Δ/Δ+*LSM1* strains (Figure 6C). These results revealed that Lsm1 plays an important role in hyphal growth and biofilm formation.

### 3.7. Deletion of LSM1 Attenuates Virulence of Candida albicans

**Figure 7:**
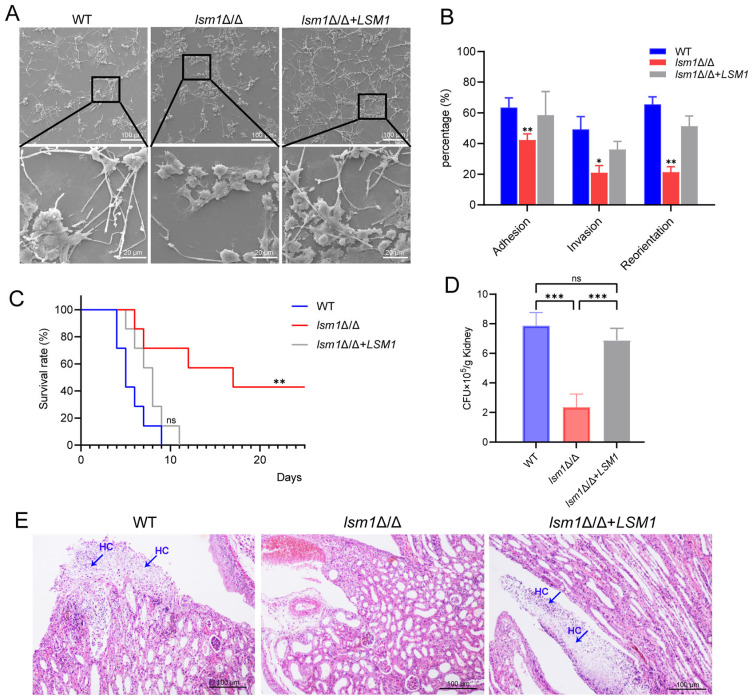
Deletion of *LSM1* attenuates virulence and pathogenicity. (**A**) In vitro *Candida albicans*-293T cell interaction model revealing the lsm1 mutant’s reduced ability to damage 293T cells, observed with a scanning electron microscope (SEM). Scale bar (**top**), 100 μm. Magnification of boxed regions at the bottom. Scale bar (**bottom**), 20 μm. (**B**) Percentage of adhesion, invasion into 293T cells, and hyphal reorientation in the *lsm1*Δ/Δ mutant. (**C**) Survival curves of mice infected by the WT, *lsm1*Δ/Δ mutant and the complemented strain *lsm1*Δ/Δ+*LSM1*. Statistical significance was determined by the Log-rank (Mantel-Cox) test. (**D**) Statistical analysis of fungal burdens in the kidneys of the injected mice. Colony-forming units (CFU) were counted (*n* = 3). (**E**) Histopathological observation of infected mouse kidneys. The blue arrows point to the hyphae invading the kidneys. Scale bar, 100 μm. Statistical significance is indicated as follows: *, *p <* 0.05; **, *p <* 0.01; ***, *p <* 0.001; ns, not significant.

To comprehensively evaluate the virulence of the strains, we utilized an in vitro *Candida albicans*-293T cell interaction model and an in vivo systemic infection model. In the in vitro model, our findings revealed that the *lsm1*Δ/Δ mutant exhibited the markedly reduced ability to damage 293T cells compared to the WT and reconstituted strains (Figure 7A). Furthermore, in the *lsm1*Δ/Δ mutant, we observed a significant reduction in adherence, invasion into 293T cells, and hyphal reorientation of the fungal cells (Figure 7B). These results revealed that the *lsm1*Δ/Δ mutant exhibited the markedly reduced ability to damage 293T cells compared to the WT and reconstituted strains. In the in vivo model, the *lsm1*Δ/Δ mutant displayed reduced virulence, with over 40% of the mice surviving past 20 days post-infection. In contrast, all mice injected with the WT strain and the reconstituted strain succumbed to the infection within 9 days and 11 days, respectively (Figure 7C). Fungal burden assays showed that the colony-forming units (CFU) in the kidneys of mice infected with the *lsm1*Δ/Δ mutant were lower than those infected with the WT strain and reconstituted strain (Figure 7D). Histopathological observation of the infected mouse kidneys revealed numerous hyphae penetrating the renal pelvic tissues in the WT strain-infected group and reconstituted strain-infected group, whereas no hyphae were detected in the *lsm1*Δ/Δ mutant-infected kidneys (Figure 7E). Collectively, these results suggest that deletion of *LSM1* attenuates the virulence of *C. albicans*.

## 4. Discussion

Fungal pathogens rapidly integrate nutritional cues with organelle homeostasis to survive and infect host tissues. In this study, we found that a subpopulation of PB is localized to mitochondria, and we characterized the function of the PB-associated protein Lsm1 in the opportunistic fungus *C. albicans*. We identified that mRNA decay factor Lsm1 participates in regulating numerous cellular processes, such as nutrient sensing, maintenance of mitochondrial function, TOR signaling, autophagy execution, hyphal growth and pathogenicity. Our findings reveal that loss of Lsm1 triggers system-wide cellular dysfunction, initiating from broad transcriptomic alterations and culminating in mitochondrial impairment, uncoupled TORC1 signaling, and defective autophagic flux. This indicates that Lsm1 is required to maintain coordinated metabolic and organelle homeostasis. Moreover, these findings suggest that post-transcriptional control is not merely a basal cellular process, but a crucial regulatory tier governing the pathogen’s stress adaptation and virulence. Despite these insights, we acknowledge certain limitations inherent in our experimental design. Although the phenotypic defects were rescued in the complemented strain, the transcriptomic profile of this strain was not assessed. Consequently, while the observed transcriptional changes are strongly associated with *LSM1* deletion, we cannot entirely exclude the potential contribution of background mutations to the global expression profile. Furthermore, given that *C. albicans* is prone to genomic rearrangements and aneuploidy, the possibility of subtle secondary genomic alterations should be considered. Additionally, while our data highlight a significant link between *LSM1* and transcript abundance, future studies employing direct measurements of mRNA decay rates will be required to formally establish the precise post-transcriptional mechanisms of Lsm1 in this context.

Our data reveal that Lsm1 is required to maintain mitochondrial function and metabolic flexibility. Mitochondria emerge as a central hub of dysregulation in *lsm1*Δ/Δ cells. Transcriptional imbalance of respiratory complexes, loss of membrane potential, and cytosolic and mitochondrial Ca^2+^ overload collectively indicate mitochondrial stress. Intriguingly, rather than undergoing oxidative collapse, *lsm1*Δ/Δ cells establish a redox balance characterized by elevated NADPH, SOD activity and GSH/GSSG ratios. Importantly, metabolic flexibility is enhanced, as growth defects are rescued when glycerol replaces glucose as the carbon source, highlighting a shift toward alternative metabolic strategies. This metabolic plasticity allows the *lsm1*Δ/Δ mutant to survive chronic mitochondrial stress but creates a fragile equilibrium. As evidenced by the rapid depletion of GSH upon rotenone exposure, *lsm1*Δ/Δ cells operate at a high metabolic cost to preserve short-term survival.

A key finding of our study is the uncoupling of autophagy induction from the degradative process in the absence of Lsm1. As an evolutionarily conserved signaling pathway, TOR is ubiquitously expressed in eukaryotic cells and serves as a critical sensor for both environmental and endogenous stress. The inhibition of TOR signaling under nutrient-limited conditions facilitates a metabolic shift from anabolic processes toward catabolic recycling to maintain homeostasis. While nutrient limitation caused by amino acid deficiency and mitochondrial dysfunction normally suppresses TORC1 activity to induce autophagy, we found that *lsm1*Δ/Δ cells display aberrant TORC1 signaling that is reduced under basal conditions but increased under nitrogen starvation. Furthermore, even when autophagy was initiated, the flux was blocked at the late stages. The accumulation of GFP-Atg8 puncta at the vacuolar membrane and the failure to generate free GFP indicate a defect in autophagosome–vacuole fusion. First, this phenotype is mechanistically consistent with the stability of *ATG* transcript levels. The Pat1-Lsm complex is known to stabilize *ATG* mRNAs during nitrogen starvation to sustain autophagic capacity; consistent with this, we observed a failure to maintain *ATG* transcript levels in starving *lsm1*Δ/Δ cells. Second, it may account for compromised endosomal–vacuolar trafficking and autophagosome–vacuole fusion, thereby uncoupling autophagy initiation from subsequent cargo degradation. This is likely because previous studies have reported interactions between P-bodies and late endosomes or multivesicular bodies in Drosophila and mammalian [35,36]. Additionally, increased commensal fitness in *C. albicans* clinical isolates has been reported to be associated with elevated TOR activity within host niches, suggesting that the TOR signaling pathway is involved in the adaptive evolution of *C. albicans* [37].

## 5. Conclusions

In summary, this study demonstrates that Lsm1 is essential for coordinating metabolic status with mitochondrial quality control and fungal pathogenicity in *C. albicans*. Our findings reveal that *LSM1* deletion significantly alters the transcriptomic landscape, particularly affecting genes involved in amino acid metabolism, mitochondrial function and autophagy. These results underscore the importance of Lsm1 in mediating the response to nutritional and oxidative stresses within the host, marking it as a potential target for antifungal therapy.

## Data Availability

The original contributions presented in this study are included in the article/Supplementary Material. Further inquiries can be directed to the corresponding authors.

## Supplementary Materials

The following supporting information can be downloaded at https://www.mdpi.com/article/10.3390/microorganisms14040771/s1, Table S1: Strains used in this study; Table S2: Primers used in this study; Table S3: Relative gene expression levels between WT and *lsm1*Δ/Δ; Figure S1: Principal component analysis (PCA) of RNA-seq samples; Figure S2: Quantitative analysis of hyphal length; Figure S3: Growth of the WT, *lsm1*Δ/Δ, and *lsm1*Δ/Δ+*LSM1* strains on YPD medium with or without 5 mM H_2_O_2_.
