# Supplementary material for: Lsm1 Coordinates Mitochondrial Homeostasis, TORC1 Signaling, and Virulence in Candida albicans
Source: Microorganisms. 2026 Mar 28;14(4):771. doi: 10.3390/microorganisms14040771 (PMC13118331; doi:10.3390/microorganisms14040771)
Supplement: Supplementary file 1 [file microorganisms-14-00771-s001.zip › microorganisms-4178266-supplementary.pdf]

Table S1 Strains used in this study

Strains	Genotype	Source
WT (BWP17)	*ura3Δ::λimm434/ura3Δ::λimm434 his1::hisG/his1::hisG arg4::hisG/arg4::hisG*	Dana Davis
*lsm1*Δ/Δ	*ura3Δ::λimm434/ura3Δ::λimm434 his1::hisG/his1::hisG arg4::hisG/arg4::hisG lsm1::ARG4/lsm1::dpl200*	This study
*lsm1*Δ/Δ+*LSM1*	*ura3Δ::λimm434/ura3Δ::λimm434 his1::hisG/his1::hisG arg4::hisG/arg4::hisG lsm1::ARG4/lsm1:: dpl200*, *LSM1*	This study
WT+URA3	*URA3/ura3Δ::λimm434 his1::hisG/his1::hisG arg4::hisG/arg4::hisG*	This study
*lsm1*Δ/Δ+URA3	*URA3/ura3Δ::λimm434 his1::hisG/his1::hisG arg4::hisG/arg4::hisG lsm1::ARG4/lsm1::dpl200*	This study
*lsm1*Δ/Δ+*LSM1*+URA3	*URA3/ura3Δ::λimm434 his1::hisG/his1::hisG arg4::hisG/arg4::hisG lsm1::ARG4/lsm1::dpl200 LSM1*	This study
WT+GFP-LSM1	*ura3Δ::λimm434/ura3Δ::λimm434 his1::hisG/his1::hisG arg4::hisG/arg4::hisG GFP-LSM1*	This study
WT+GFP-ATG8	*ura3Δ::λimm434/ura3Δ::λimm434 his1::hisG/his1::hisG arg4::hisG/arg4::hisG GFP-ATG8*	This study
*lsm1*Δ/Δ+GFP-ATG8	*ura3Δ::λimm434/ura3Δ::λimm434 his1::hisG/his1::hisG arg4::hisG/arg4::hisG lsm1::ARG4/lsm1::dpl200 GFP-ATG8*	This study
*lsm1*Δ/Δ+*LSM1*+GFP-ATG8	*ura3Δ::λimm434/ura3Δ::λimm434 his1::hisG/his1::hisG arg4::hisG/arg4::hisG lsm1::ARG4/lsm1:: dpl200*, *LSM1*, *GFP-ATG8*	This study
WT+SCH9-HA	*ura3Δ::λimm434/ura3Δ::λimm434 his1::hisG/his1::hisG arg4::hisG/arg4::hisG SCH9-HA*	This study
*lsm1*Δ/Δ+SCH9-HA	*ura3Δ::λimm434/ura3Δ::λimm434 his1::hisG/his1::hisG arg4::hisG/arg4::hisG lsm1::ARG4/lsm1::dpl200 SCH9-HA*	This study
*lsm1*Δ/Δ+*LSM1*+SCH9-HA	*ura3Δ::λimm434/ura3Δ::λimm434 his1::hisG/his1::hisG arg4::hisG/arg4::hisG lsm1::ARG4/lsm1:: dpl200*, *LSM1*, *SCH9-HA*	This study
**Plasmids**		
pDDB78	Amp^R *TRP1 HIS1*	Dana Davis
pDDB78-LSM1	Amp^R *TRP1 HIS1 IPK1*	This study
pRS-ARG4Δ*Spe*I	Amp^R *ARG4*	Dana Davis
pDDB57	Amp^R *URA3*	Dana Davis
pAU34M-GFP-LSM1	Amp^R *URA3* P_{ACT1}*-GFP-LSM1*	This study
pAU34M-GFP-ATG8	Amp^R *URA3* P_{ACT1}*-GFP-ATG8*	This study
pLUBP	Ap^R *URA3* (*in vivo*)	William A. Fonzi

Table S2 Primers used in this study

Name	Sequence (5′ -3′)
LSM1-5DR	AATCAAAAAAAAAAACCACACAACAGAAACACCCAACGATAG GAAATAACTACATATTTTATTTCCCAGTCACGACGTT
LSM1-3DR	AACTGCTTTGTCTTTACTTTTTGTTCTCTGATCTTTTTTGCTTGT TGTTCTTTCAACTCATGGAATTGTGAGCGGATA
LSM1-5det	GTGAATAATCGGTATAGCAAAA
LSM1-3det	AACATGGAAACTCAACGTAGCA
LSM1-5com	GACTAGTCCCAATCATTTTATCATGC
LSM1-3com	CGGGGTACCTTATGGCTGGTGTTGGTAG
5GFP-LSM1	TCCCCCGGGTCAAGTGTATCAT
3GFP-LSM1	CGCGGATCCTTAATACAAATCT
ATG1-5RT	ACGAGTCCTGTACCAGACGA
ATG1-3RT	TTGCGTGAGTTTGGAATGGC
ATG2-5RT	CACCAGGAAGGTTGAGCTGT
ATG2-3RT	GGCGAGTAGCAGATGACGAA
ATG3-5RT	GACCCCTTAGATCGATTCAT
ATG3-3RT	GGTTTCCAAAGTTTACGTGA
ATG4-5RT	CGCGGGATTTGGATGGTAT
ATG4-3RT	TCTTGGCTCTTGGTTGGTG
ATG5-5RT	GCACTGCCAAACCAATAATGC
ATG5-3RT	CACAATGGGAGATCCGGCAA
ATG8-5RT	CAGCCAGAATTGCTCAGAGGT
ATG8-3RT	TGGCTTTTTCGCTTGGTAACT
ATG9-5RT	GCTGCCCCTATGGATTTGTC
ATG9-3RT	CGACAGTCCCTGATTCTCCG
ATG11-5RT	GCTGAGGAATCCATCCACCA
ATG11-3RT	TGATCTGATTTGTGAGCTCGGT
ATG13-5RT	GTACGTCTGTTCCACGGTCT
ATG13-3RT	TTGCGCGTGAACCAAATGAC

Table S3 Relative gene expression levels between WT and *lsm1*Δ/Δ

Gene name	Log(fold change)	Percent (%)	Gene name	Log2(fold change)	Percent (%)
ALT1	-0.26928	-17.0	*QDR3*	-0.16412	-10.8
PYC2	-0.50196	-29.4	*GNP2*	0.362024	28.5
ASN1	-0.26741	-16.9	*GNP1*	-0.37333	-22.8
HOM2	-1.14858	-54.9	*AGP2*	0.419758	33.8
HOM3	-0.54741	-31.6	*AGP3*	0.24982	18.9
HOM6	-0.34891	-21.5	*DIP5*	0.378406	30.0
THR4	-0.49606	-29.1	*DIP53*	2.162391	347.7
CHA1	-0.32697	-20.3	*AVT42*	0.840128	79.0
ILV5	-0.26532	-16.8	*AVT4*	0.353062	27.7
ILV6	-0.34475	-20.3	*MTR1*	0.271286	20.7
ILV1	-0.38414	-23.4	*NDE1*	-0.64322	-36.0
ILV2	-0.42927	-25.7	*FESUR1*	0.34079	26.6
ILV3	-0.57894	-33.1	*ALI1*	0.324597	25.2
BAT22	-0.446	-26.6	*MCI4*	0.351966	27.6
BAT21	-0.27321	-17.3	*SDH1*	0.126668	9.2
LEU4	-0.41885	-25.2	*SDH2*	-0.38677	-23.5
LEU42	-0.50657	-29.6	*SDH3*	-0.40079	-24.3
GLN1	-0.57466	-32.9	*SDH4*	-0.39801	-24.1
ARG5	-0.2819	-17.7	*COB*	-0.55054	-31.7
ARG8	-0.32186	-20.0	*CYC1*	-0.1864	-12.1
ARG3	-0.67279	-37.3	*QCR10*	0.167645	12.3
ARG1	-1.33645	-60.4	*QCR8*	-0.76748	-41.3
PRO1	-0.2934	-18.4	*QCR9*	0.05938	4.2
LYS9	-0.2796	-17.6	*QCR7*	0.41679	33.5
LYS21	-0.30005	-18.8	*CYT1*	-0.2988	-18.7
LYS12	-0.33319	-20.6	*COX4*	-0.37387	-22.8
LYS2	-0.42247	-25.4	*COX5*	0.412138	33.1
LYS4	-0.45808	-27.2	*COX9*	-0.68852	-38.0
LYS22	-0.49808	-29.2	*COX11*	-0.50888	-29.7
LYS1	-0.74403	-40.3	*ATP14*	0.25976	19.7
HIS4	-0.53039	-30.8	*ATP16*	-0.45739	-27.2
HIS7	-0.32218	-20.0	*ATP18*	0.236926	17.8
ARO4	-0.49238	-28.9	*ATP20*	0.488295	40.3
ARO2	-0.64632	-36.1	*PFK1*	-0.26709	-16.9
ARO1	-0.36532	-22.4	*FBA1*	-0.30569	-19.1
ARO3	-0.34051	-21.0	*TDH3*	-0.26713	-16.9
ARO8	-0.69464	-38.2	*TPI1*	-0.38232	-23.3
TRP2	-0.4511	-26.9	*PGK1*	-0.38163	-23.2
TRP4	-0.39162	-23.8	*ENO1*	-0.37781	-23.0
TRP5	-0.40408	-24.4	*TKL1*	-0.46153	-27.4
SER33	-0.58405	-33.3	*RPE1*	-0.27503	-17.4
SER1	-0.38397	-23.4	*PRS4*	-0.33473	-20.7
CYS4	-0.40724	-24.6	*PRS1*	-0.26095	-16.5
CYS3	-0.38914	-23.6	*TAL1*	-0.4589	-27.2
MET2	-0.26991	-17.1	*FBP1*	0.149675	10.9
MET15	-0.28106	-17.7	*PCK1*	0.122764	8.9
STR3	-0.31278	-19.5	*ICL1*	-0.48936	-28.8
MET6	-0.59346	-33.7	*FOX2*	-0.03079	-2.1
SAM2	-0.39628	-24.0	*MLS1*	0.026232	1.8
SHM1	-0.34097	-21.0	*GUT1*	0.649032	56.8
SHM2	-0.43745	-26.2	*GUT2*	0.096327	6.9
GLY1	-0.41421	-25.0	*ACO1*	-0.41503	-25.0
AQR1	-0.17423	-11.4	*ACO2*	-0.33442	-20.7
QDR2	-0.16628	-10.9	*IDP1*	-0.30046	-18.8

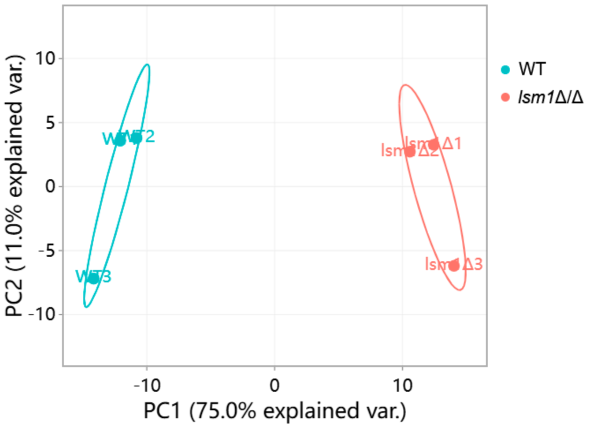

Figure S1. Principal component analysis (PCA) of RNA-seq samples. PCA was performed based on normalized gene expression data from wild-type and *lsm1*Δ/Δ strains. Each point represents an individual biological replicate.

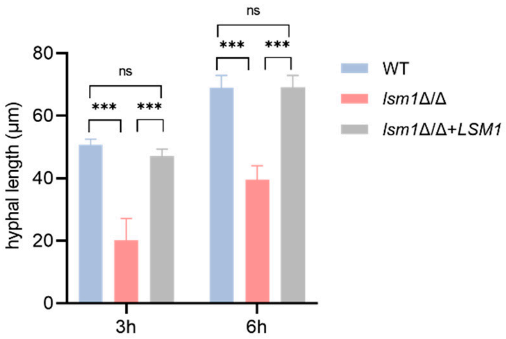

Figure S2. Quantitative analysis of hyphal length. Data are presented as mean ± SEM from three independent experiments (n>50 hyphae per strain). Asterisks indicate statistical significance determined by One-way ANOVA followed by Tukey's post-hoc test (***p<0.001; ns, non-significant).

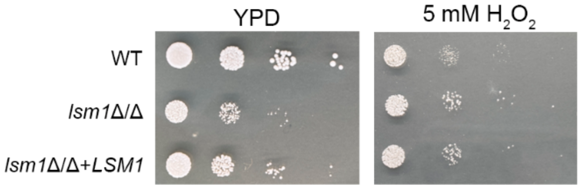

Figure S3. Growth of the WT, *lsm1*Δ/Δ, and *lsm1*Δ/Δ+*LSM1* strains on YPD medium with or without 5 mM H_2O_2.
